# A Multi-Sensor Cane Can Detect Changes in Gait Caused by Simulated Gait Abnormalities and Walking Terrains

**DOI:** 10.3390/s20030631

**Published:** 2020-01-23

**Authors:** Satinder Gill, Nitin Seth, Erik Scheme

**Affiliations:** 1 Institute of Biomedical Engineering, University of New Brunswick, Fredericton, NB E3B 5A3, Canada; satinder.gill@unb.ca (S.G.); nitin.seth@unb.ca (N.S.); 2 Department of Electrical and Computer Engineering, University of New Brunswick, Fredericton, NB E3B 5A3, Canada

**Keywords:** multi-sensor, assistive technologies, cane, gait, proactive monitoring, inertial measurement unit (IMU), simulated gait abnormalities, walking terrain, vision, cane length

## Abstract

Due to the increasing rates of chronic diseases and an aging population, the use of assistive devices for ambulation is expected to grow rapidly over the next several years. Instrumenting these devices has been proposed as a non-invasive way to proactively monitor changes in gait due to the presence of pain or a condition in outdoor and indoor environments. In this paper, we evaluated the effectiveness of a multi-sensor cane in detecting changes in gait due to the presence of simulated gait abnormalities, walking terrains, impaired vision, and incorrect cane lengths. The effectiveness of the instrumented cane was compared with the results obtained directly from a shank-mounted inertial measurement unit. Results from 30 healthy participants obtained while simulating gait abnormalities and walking over different terrains demonstrated the ability of the cane to reliably and effectively discriminate among these walking conditions. Moreover, the results obtained while walking with impaired vision and incorrect cane lengths indicate the ability of cane to detect changes in gait during these scenarios as well.

## 1. Introduction

Globally, healthcare systems are being stressed as a result of the aging baby-boomer demographic and the rise of chronic diseases and neurological impairments [1,2]. Among a variety of increased medical complications, mobility impairments due to reduced coordination, muscle strength and postural balance are a major source of concern in these populations. In order to combat this, there has been increased focused on developing innovative proactive solutions that enable self-management and early intervention to alleviate the burden on healthcare providers and caregivers [3]. One proactive solution is to monitor people’s behaviors and mobility during everyday life as opposed to performing controlled and reactive assessments in the context of a lab or specialist clinic [4,5].

The gold standard for mobility and gait monitoring most often includes full-body motion capture systems, video systems, and force places that are capable of providing highly detailed and quantitative gait data [6,7]. These systems, however, are often prohibitively expensive for most clinical settings, require the need for additional skilled technicians, and are limited to indoor use in laboratory settings [8,9,10]. Moreover, these solutions can only provide gait data for a limited number of strides thus preventing them from being a viable option for community-based, long-term monitoring.

To address these challenges, wearable sensor technology provides greater flexibility by allowing for consistent monitoring of regular everyday activities across multiple indoor and outdoor environments. Wearable motion sensors, such as inertial measurement units (IMUs) which combine accelerometers and gyroscopes, have been widely researched for gait monitoring in elderly subjects and pathological populations [5,8]. A strength of these low power systems is their small size, allowing them to be placed almost anywhere on the body as a single standalone, multi-channel, or even a multi-sensor system [9,11,12]. Their data provide enough detailed information to extract relevant gait characteristics including step length, number of steps, stride variability, and walking speed [12]. However, there are some limitations that have restricted widespread adoption of these technologies among the populations that could benefit the most from their use. Firstly, there are concerns related to adoption and adherence due to the lack of technological knowledge and/or cognitive ability [11,12]. Secondly, there are concerns related to the obtrusiveness of these devices and opposition to behavioral change [13]. 

To address these challenges while maintaining the ability to monitor in everyday environments, the assistive gait aids (ADs) that these populations often already use have been instrumented [14,15,16]. Recent studies suggest that approximately 6.1 million community-dwelling American adults ambulate with the support of an AD to combat mobility impairments and mitigate instability [17]. This has led researchers to develop instrumented ADs to help monitor and categorize gait activity [16,18,19]. These works the presented mechanical design of instrumented canes equipped with IMUs and a load monitoring system intended to be used as diagnostic tools in physiotherapy clinics. The work presented in References [18,19] used load cells to measure axial loading information, whereas Wade et al. [16] used force-sensing resistors incorporated into the cane handle and base to measure loading information. While these early works demonstrated the potential of this approach, limitations including cost, reliability, and lack of industrial design have prevented their successful deployment outside of training situations [14]. In our previous works [14,15], the potential to perform preventative gait and mobility monitoring using affordably instrumented gait aids was demonstrated by leveraging the Internet of Things (IoT) principles to create “smart” technologies. Using this approach, accurate gait segmentation has been shown to be possible across different walking terrains [20]. 

It has been shown that an instrumented wheeled walker can also be used to reflect changes in gait during rehabilitation and predict the outcomes of clinical assessments [21]. Presented results revealed the ability of an instrumented walker to predict Tinetti assessment scores (measure of balance and gait function) on the fly. Clinician provided scores were compared to scores predicted by an instrumented walker and showed a small mean square prediction score. In our previous work, instrumented walkers have also demonstrated the ability to determine both the activity level of the individual and the type of environment in which they are walking [15]. Incident monitoring has also been demonstrated to be possible with instrumented walking canes that are capable of detecting falls [22,23]. For example, Lan et al. [23] implemented a fall detection algorithm based on subsequence matching by recognizing three stages of a fall pattern. Four types of falls (i.e., forward, backward, side, and free fall) were simulated using healthy participants. The presented results indicated the ability of the algorithm to detect the majority of the falls while achieving very low false-positive rates for non-falling conditions. While these works performed well in fall detection, they provide evidence only for situations where someone has already fallen as opposed to the preventative monitoring of near-falls or altered gait patterns. To detect subtle changes in gait, Wade et al. [16] presented an alternative instrumented cane design that could identify when an individual was walking on stairs, walking with their eyes closed, as well as walking while looking to one’s side. The results from seven adult volunteers showed overall accuracy of 95.8% in classifying these activities using force and inertial information collected from the cane’s sensors. Recently, the relationship between user and cane movement was investigated to provide clinicians and caregivers insightful information about cane-assisted walking [24]. Gyroscope data from shank-mounted and cane IMUs were collected for two participants undergoing post-stroke rehabilitation during a continuous standardized sequence of ambulation activities. Presented results revealed that shank-mounted and cane IMUs can capture relevant information related to a user’s gait. In our previous works, we have demonstrated the potential of a multi-sensor cane to detect changes in gait due to the fact of pain-related simulated gait perturbation [14]. To truly enable preventative monitoring and intervention, however, these devices must all be able to function robustly during an individual’s everyday life activities. This requires the ability to detect changes in gait caused due to the presence of pain, injury, changes in walking terrains, or visual distractions. 

In this work, we examined the potential of a multi-sensor cane to act, not only as an activity monitor, but also as a tool to relay information about the nature of the user’s gait. To this aim, we sought to determine the capabilities of an instrumented cane in detecting a variety of gait-related changes as validated by a shank-mounted IMU. Specifically, the desired outcomes included the ability to identify simulated changes in gait, walking terrain, simulated vision perturbations, and incorrect cane length. Improvements in these factors may help validate the instrumented multi-sensor cane as an important deployable tool for preventative monitoring of vulnerable individuals as they perform their everyday activities and routines. 

## 2. Materials and Methods

### 2.1. System Overview

**Figure 1:**
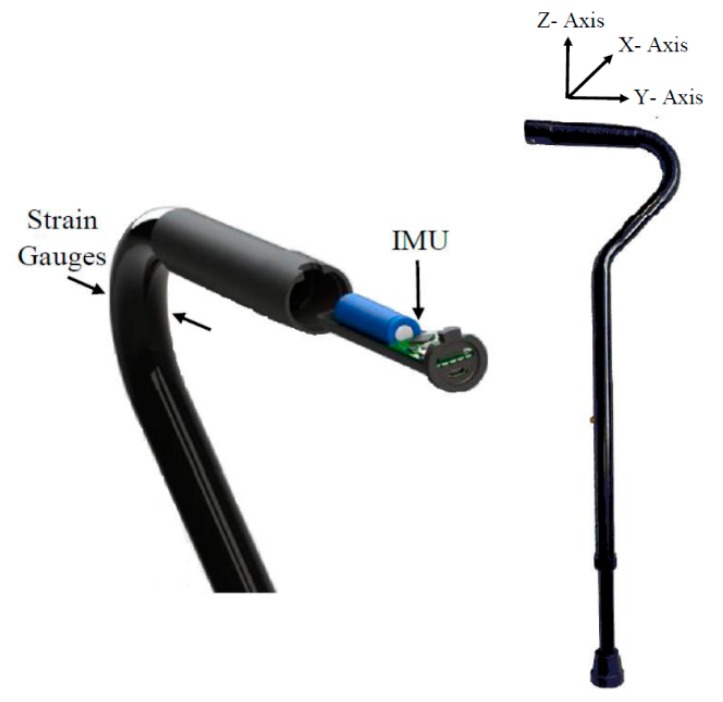
Multi-sensor Internet of Things (IoT) enabled cane [14,20]. IMU—inertial measurement unit.

The system used in this work consisted of an IoT-enabled, multi-sensor cane developed by the Health Technologies Laboratory and Institute of Biomedical Engineering at the University of New Brunswick, Fredericton, Canada. The system design and implementation are described in detail in previous works [14]. The key requirement for the system design was that it was nearly indistinguishable from a standard offset cane. Therefore, a 3D printed carriage housing the system electronics was designed to fit the handle of a standard commercially available offset cane. Figure 1 shows a rendered model (left) and the final design (right) of the multi-sensor cane used in this work. The multi-sensor cane is capable of measuring load exerted on the cane as well as motion and orientation data. The loading information was measured using strain gauges (SGT-1A/1000-TY13, Omega Engineering) arranged in Wheatstone topology. Two strain gauges were fixed on the apex of the multi-sensor cane’s curvature while other two were mounted to a matching piece of aluminimum for temperature compensation. A 9-axis inertial measurement unit (LSM9DS1), consisting of accelerometer, gyroscope, and magnetometer was set to provide angular velocity and acceleration signals with a sensitivity of 500 °/s and 16× *g* respectively. All sensors were sampled at a frequency of 231 Hz. The multi-sensor cane can either store measured data on a microSD card or use Bluetooth Low Energy (BLE) to streamline data to a nearby tablet. For this experiment data were stored locally on miscroSD card for offline processing. We used two coordinate systems: the IMU coordinate system and a global Cartesian system. The *z*-axis of the global Cartesian system is in parallel with the gravitational acceleration, and the XY plane is assumed to be level and perpendicular (i.e., user is on flat ground) with the *y*-axis pointing in the walking direction. The axes of the IMU coordinate system coincided with the axes of the global Cartesian system. 

### 2.2. Experimental Protocol

Thirty healthy individuals (24 male and 6 female, aged 18–31 years, mean ± SD = 22.0 ± 3.1 years) with no known injuries participated in this study. The cane length was adjusted to be an appropriate height for each individual [25]. For trials that involved improper cane height, the cane’s length was adjusted to be 2 inches too tall or 2 inches too short. Prior to walking, all participants received a brief tutorial on cane-assisted gait (https://www.youtube.com/watch?v=fRn8ZZJMzno) as well as a practice session to familiarize themselves with walking with an assistive gait aid. The experiment only proceeded once the participant was comfortable enough to use the cane correctly while walking while also being able to engage in conversation. For each of the testing conditions, listed in Table 1, each participant was instructed to walk at a self-selected speed with the help of the cane. Each individual performed walking trials representing all 11 walking conditions. All subjects provided written informed consent prior to participating. This study was approved by the University of New Brunswick Research Ethics Board (REB #2017-097).

### 2.3. System Validation

**Figure 2:**
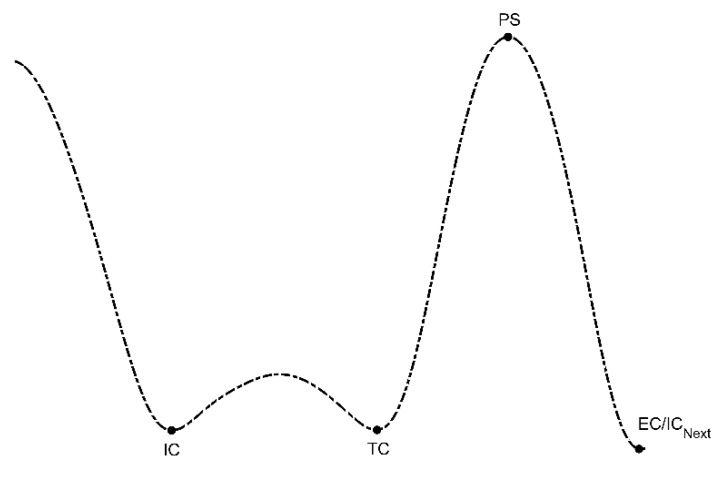
Initial contact (IC), terminal contact (TC), peak swing (PS), and end of contact (EC) events corresponding to gyroscope data for shank-mounted IMUs.

To validate that the data from the multi-sensor cane provided accurate data relevant to gait, a Shadow full-body inertial motion capture system (Motion Workshop) was used. The Shadow system consists of 18 body-mounted sensors, sampling at 100 Hz, that are placed along an individual’s limbs to track the entirety of the body’s movement. Of the 18 sensors, three sets of sensors served as potential validation candidates: sensors placed within the shoe insoles (kinetic information), sensors placed on the shoes, and shank-mounted IMU sensors (kinematic information). Sensors placed in shoe insoles can provide gait-related kinetic information; however, they often suffer from wear and tear and are also influenced by the drift typically observed with force-sensing resistors (FSRs). Shoe-mounted IMU sensors can provide kinematic information related to movement; however, these sensors suffer from motion artifacts caused from the heel strikes during walking. In this study, shank-mounted IMU sensors were used to obtain kinematic information, as these sensors are less affected by motion artifacts and have been widely used in the literature [24,26]. All Shadow system data were collected and stored for offline processing using MATLAB^®^ (Mathworks Inc., Natick, MA, USA). High-frequency motion artifacts and noise components were removed from these shank-mounted sensors using sixth-order, low-pass filters with cutoff frequencies of 8 Hz. A threshold-based gyroscope peak detection (GPD) algorithm similar to the one presented in Reference [24] was developed and used to segment continuous gait data from the Shadow system into individual strides. The algorithm operated on shank medial–lateral (*x*-axis) gyroscope data. This is referred to as shank anteroposterior (AP) velocity. To ensure accurate baseline results, a specific optimal threshold value was selected for each participant per condition based on manual inspection of shank gyroscope data. This is explained in detail elsewhere [20,24]. Briefly, peaks in gyroscope AP velocity above threshold value are classified as peak swing (PS) events. Thereafter, initial contact (IC) events corresponding to a local minimum after PS events are identified. Similarly, terminal contact (TC) events that corresponds to local minimum before PS events are detected. Lastly, end of contact (EC) events that correspond to local minimum after PS events are identified. During continuous walking, EC event corresponds to the IC event of the next gait cycle. This is demonstrated in Figure 2. In this fashion, all valid strides and corresponding temporal events (i.e., IC, TC, PS, and EC) were identified using the Shadow system’s shank-mounted IMU.

### 2.4. System Data Segmentation

The raw data from the cane’s sensors were collected and stored from each participant for offline processing using MATLAB^®^ (Mathworks Inc. Natick, MA, USA). High-frequency motion artifacts and noise components were removed from both sets of sensors using sixth-order, low pass filters with 4 and 8 Hz cutoff frequencies for strain gauge and IMU sensor data, respectively. Continuous gait data were segmented into the individual strides for every tested condition. Relevant walking cycle events, such as initial contact (IC), terminal contact (TC), peak swing (PS), and end of contact (EC), were identified. Of note, the IC, TC, and EC events all corresponded to zero-crossings in the strain gauge data. By contrast, the PS event corresponded to a peak in the multi-sensor cane (*x*-axis) gyroscope data. This is called the cane AP velocity. Segmentation and walking-cycle-event identification were both performed using a multi-sensor matched filter (MSMF), a template matching algorithm, described in detail in our previous work [20]. Briefly, the walking cycle is categorized into two phases: the cane-loading phase and the cane-swing phase. The loading phase begins once the IC is detected and continues as the cane touches the ground and the user steps forward while loading the cane for support. The IC event is identified by correlating a known template signal with the unknown strain gauge data. A similarity threshold of 50% was used to avoid spurious positive peaks in the matched filter output being identified as valid strides. The IC events were identified as positive strain gauge zero-crossing points within 0.5 s of the matched filter’s positive peak to account for varying stride lengths. In order to directly compare with this MSMF approach, the GPD approach used with the Shadow system was also applied to the cane’s gyroscope data to extract the IC event.

The loading phase ends with the TC event, as the user gets ready to lift the cane off the ground. The TC event was identified as the negative strain gauge zero-crossing point following the IC event. This is followed by the swing phase during which the user swings the cane in the anteroposterior direction. The location of the peak, or maximum, value in the gyroscope AP velocity following a TC event was identified as the PS event. 

**Figure 3:**
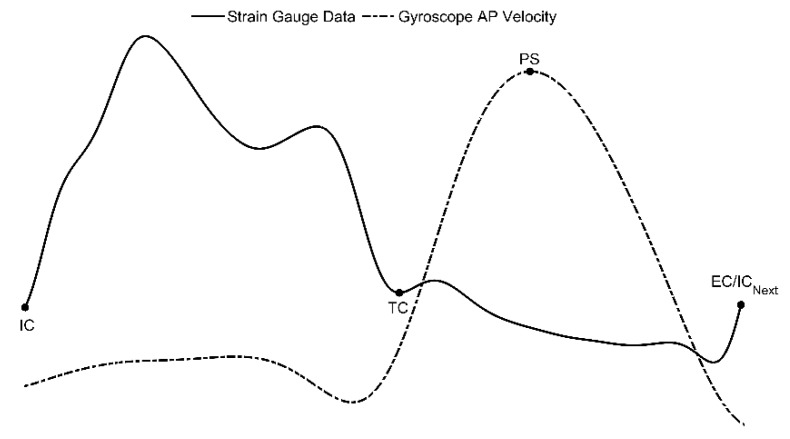
IC, TC, and EC events corresponding to strain gauge data and PS event corresponding to gyroscope AP velocity for multi-senor cane.

Lastly, the EC event corresponds to an instant at the end of the swing phase once the user stops swinging the cane and places the cane-tip in front of themselves. The time interval between IC and EC events here defines a full gait cycle. The EC event was identified as the positive strain gauge zero-crossing point that follows the PS event. Figure 3 shows an example of a gait cycle for multi-sensor cane data. All valid strides and corresponding temporal events (i.e., IC, TC, PS, and EC events) were identified in a similar fashion.

### 2.5. System Syncronization

Both the multi-sensor cane and shadow system started at times independent of each other and collected data at different sampling rates. In order to align the starting points for both these systems, participants were instructed to stand still while holding the cane off the ground for half a minute before starting each walking trial. A first positive strain gauge zero-crossing point and first local minima in gyroscope AP velocity of shank-mounted IMU were deemed as starting points for both the cane and shadow system, respectively. Similarly, at the end of walking trial, participants were instructed to stand still while holding the cane off the ground. The same procedure was followed for every walking trial. This allowed us to determine the starting and stopping points for both systems during each walking trial.

### 2.6. System Evaluation

System evaluation conducted to ensure that the multi-sensor cane was capable of capturing relevant gait information. Specifically, data from normal walking was compared to simulated perturbed walking conditions due to pathology, terrain, vision, or improper cane lengths. A set of relevant features that quantify gait were extracted from various cane sensors. Shown in Table 2, the features were extracted from the cane data to quantify both how the cane moved and how much it was relied upon during the various walking conditions. To assess whether the features were relevant in capturing consistent and relevant information in the strides, a repeated measures ANOVA was performed using MATLAB with a significance threshold of 0.05. To determine if differences among the conditions were present in the features characterizing the sensor data, a multiple comparison of means across conditions was performed with Bonferroni correction for each of the features listed in Table 2.

## 3. Results

Four separate experiments were conducted to determine the ability of the multi-sensor cane to determine changes in gait caused by simulated gait abnormalities, changes in walking terrain, simulated vision perturbations, and incorrect cane length. The results of the four experiments are presented here to validate the ability of the multi-sensor cane to distinguish the various walking conditions from normal walking (control case). 

### 3.1. Gait Abnormalities

Features were extracted from the cane’s sensor data and subsequently compared statistically. A Bonferroni comparison tested for differences among features extracted from the different gait conditions. These results, listed in Table 3, demonstrate that the multiple features that yielded significantly different (*p* < 0.05) means were plantarflexed and dorsiflexed gait with respect to the healthy control case. Moreover, maximum pitch velocity during both the swing and stance phases; as well as stride length denoted a significant difference for the dorsiflexion with respect to plantarflexion condition.

**Figure 4:**
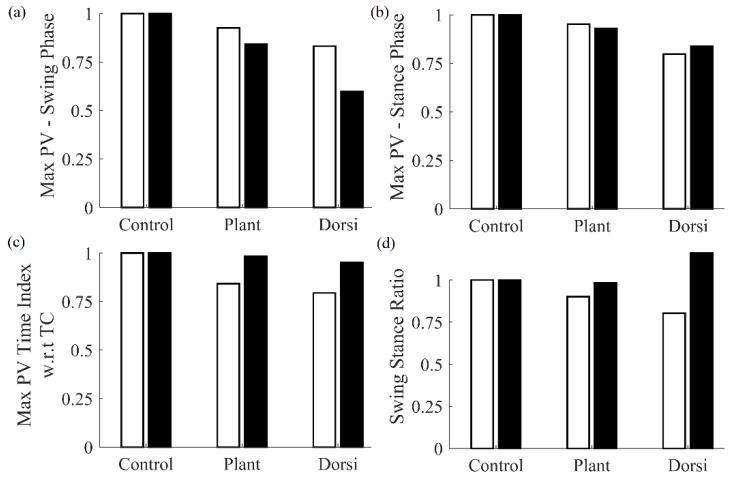
Comparison of features extracted using the cane (white bar) and shank-mounted IMU (black bar) for simulated gait abnormalities. Each feature was normalized with respect to the control condition for the cane and shank IMUs, respectively.

**Figure 5:**
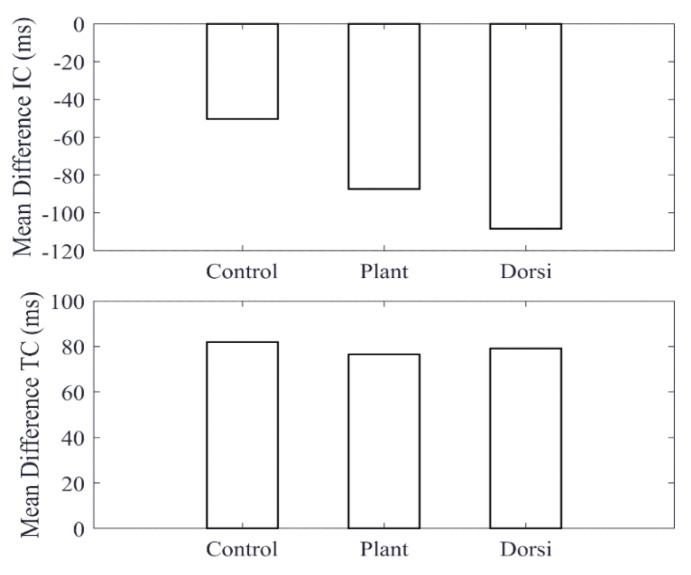
Mean difference in IC and TC events as determined by the MSMF and GPD algorithms using the cane data (IC_MSMF_ – IC_GPD_ and TC_MSMF_ – TC_GPD_).

A comparison of features obtained from the cane and the shank-mounted IMU are shown in Figure 4. All features from the cane and shank-mounted IMU follow the same trend of decreasing in value with respect to the control condition. The exception was found to be the “swing stance ratio” feature (Figure 4d). By inspection, the cane data indicate that this was smaller for dorsiflexion with respect to the control case, whereas the shank-mounted IMU data indicate an increase. This can be explained due to the difference in the IC and TC events determined by the MSMF and GPD segmentation algorithms as shown in Figure 5. A negative difference in the IC suggests that the GPD algorithm determined IC events later in time, whereas a positive difference in TC means that GPD determined the TC event to occurs earlier.

### 3.2. Walking Terrains

Table 4 lists the results of a Bonferroni comparison of the differences in features for uphill and downhill walking with respect to level walking. The differences in upstairs and downstairs walking with respect to level walking are presented in Table 5.

When walking on uphill or downhill terrains, pitch velocity during the swing and stance phases was higher with respect to the control case, and no significant difference was observed in the swing stance ratio. By contrast, while walking up and down stairs, pitch velocity during the swing and stance phases was lower with respect to the control and a significant decrease in swing stance ratio was observed. 

For all terrains, maximum strain increased with respect to the control case. In addition, stride length slightly decreased for uphill and downhill walking, whereas a significant decrease in stride length was observed for upstairs and downstairs walking. Lastly, no significant difference in mean difference in IC as determined by the MSMF and GPD approaches was observed for downhill walking, whereas a significant difference was observed for uphill walking. For upstairs and downstairs walking, a significant difference in IC determined by these approaches was observed with respect to control case. For some features, significant differences were observed in downhill walking with respect to uphill walking. When comparing downstairs walking with respect to upstairs walking, significant differences were observed for maximum pitch velocity during swing phase, stride length, and difference in IC determined by the two approaches.

**Figure 6:**
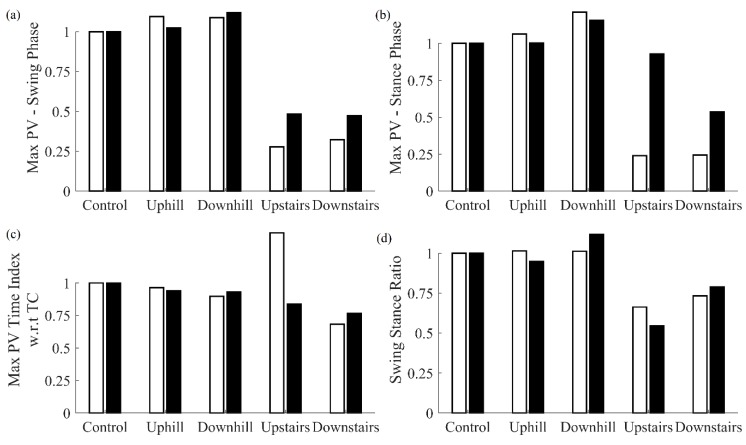
Comparison of features extracted using cane and shank-mounted IMU for walking terrains. Each feature was normalized with respect to the control condition for cane and shank IMUs, respectively (cane: white bar, shank IMU: black bar).

Figure 6 shows a comparison of features obtained for various walking terrains for the cane and shank-mounted IMU. All features for both follow the same trend with respect to control case except for Max PV Time Index with respect to TC. 

### 3.3. Impaired Vision

Next, we investigated the ability of the cane to detect any changes in gait due to the fact of impaired vision. The results presented in Table 6 show that swing stance ratio for the impaired vision conditions saw significant differences with respect to the control case. Maximum strain increased significantly for impaired vision conditions with respect to the control case. Moreover, the mean difference in IC, as determined by the MSMF and GPD approaches, increased significantly for impaired vision conditions with respect to the control case.

Maximum pitch velocity during both the swing and stance phase, as well as stride length, decreased significantly for both eyes closed conditions. No significant differences in these features were observed for the fogged glasses case with respect to the control case. 

**Figure 7:**
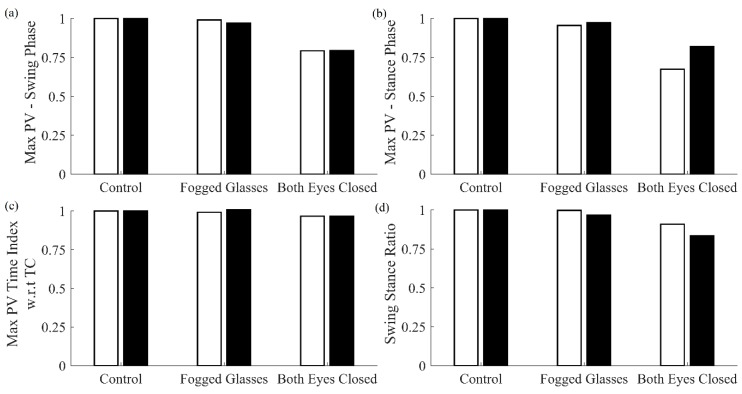
Comparison of features extracted using cane (white bar) and shank-mounted IMUs (black bar) for perturbed vision conditions. Each feature was normalized with respect to the control condition for cane and shank IMUs, respectively.

Results for the comparison of features for both cane and shank-mounted IMU are presented in Figure 7. As can be seen, the results obtained using shank-mounted IMUs follow similar trends as those obtained using the cane. 

### 3.4. Incorrect Cane Length

Lastly, the cane’s ability to detect changes in gait due to the incorrect configuration of the cane length was investigated (Table 7). Users were found to rely more on the cane for incorrect cane lengths. Intuitively, stride length increased while walking with a longer cane, whereas the opposite trend was observed for a shorter cane. An increased mean difference in IC, as determined by MSMF with respect to GPD, indicates a change in walking pattern due to the incorrect cane length. No significant difference was observed in the other features for the shorter cane case. For the longer cane condition, significant difference was observed in the swing stance ratio.

**Figure 8:**
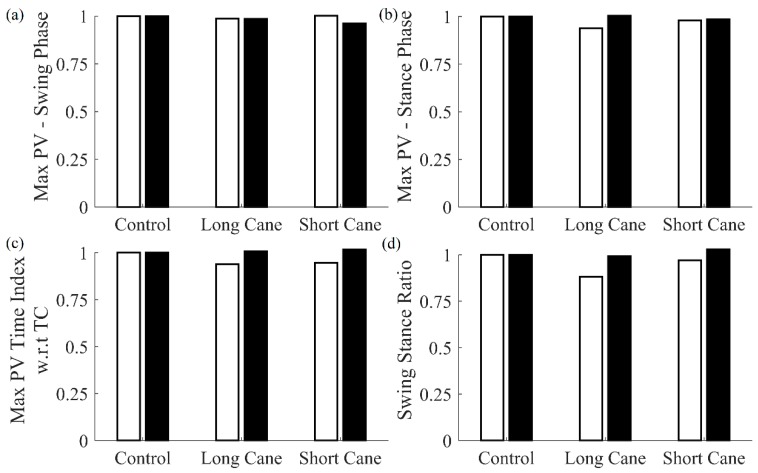
Comparison of features extracted from the cane (white bar) and shank-mounted IMUs (black bar) for perturbed cane length. Each feature was normalized with respect to the control condition for cane and shank IMUs, respectively.

Results of the comparison between the features extracted by the cane and shank-mounted IMU follow similar trends (see Figure 8).

## 4. Discussion

Proactive monitoring and intervention may help to alleviate the growing demands on global healthcare systems. This will require new and innovative solutions that can capture relevant information about patients without intruding on their quality of life. Instrumenting existing assistive devices that a person already uses may be an effective method of capturing changes in gait caused due to the fact of pain, walking terrains, altered vision, etc. in a minimally invasive way. This is important as changes in gait, particularly in response to pain originating from chronic disease, are known to be associated with reduced mobility, disability, and quality of life. Decreases in the level of activity or avoiding different walking terrains, such as stairs, can also be indicative of a person’s health. Similarly, changes in vision caused due to the fact of aging may also be an indicator of health. Improper configuration of assistive device settings can result in bad posture, potentially exacerbating problems and further impacting quality of life. Recognizing negative trends and differences in activity levels in daily life may provide vital information that could allow proactive monitoring of an individual’s well-being. Early interventions from healthcare professionals, especially for aging populations, may help reduce incidents and enable aging in place. 

The design of the tested multi-sensor cane provides a low-cost method for detecting deviations from healthy walking patterns without requiring clinical intervention or assessment. In this study, the cane consistently demonstrated the ability to detect changes in gait due to the presence of perturbed gait. The mean differences observed between healthy controls and perturbed gait conditions indicate the device can identify changes from a baseline state. Reduced pitch velocity during stance and swing phases indicate that users tended to swing their cane more slowly for perturbed gait conditions. Smaller values of maximum pitch velocity index with respect to the TC event indicate that users reached their maximum velocity earlier for perturbed gait conditions, possibly suggesting apprehension towards the end of the stride. Similarly, small values of swing stance ratio indicate that users tended to spend more time in stance phase for perturbed gait conditions. This is likely due to the lower stability and confidence. Higher values of maximum strain also indicate that the users relied more on the cane while walking under perturbed gait conditions. Moreover, the negative mean difference in maximum strain index with respect to the IC event for the stance phase indicates that users reached their peak load later in stance phase for perturbed gait conditions. It can also be seen that stride length significantly decreased for perturbed gait conditions. Lastly, the mean difference in IC event, determined by taking the difference between the IC events determined by the MSMF and GPD approaches, increased for perturbed gait. Together, this collection of metrics may be used to build indicators of changes in gait due to the presence of pain or other factors. 

Changes in gait due to the fact of walking terrains were also detectable by the multi-sensor cane. Specifically, the swing and stance phases demonstrated higher values of pitch velocity for uphill and downhill terrains with respect to the control case. This finding suggests that the users were swinging their canes faster while on these terrains. By contrast, while walking on stairs, a reverse trend in pitch velocity during swing and stance phase was observed for both upstairs and downstairs terrains. This indicates that users tended to swing their cane slower while encountering stairs. In addition, smaller values of swing stance ratio for stair-walking indicates that users tended to spend more time in stance phase. As observed during the perturbed gait conditions, users again tended to rely more on their canes for different walking terrains. The mean difference in IC events determined by the MSMF and GPD algorithms increased for all walking terrains except for downhill walking, indicating that changes in the walking pattern occurred due to the different terrains.

Changes in gait due to the fact of reduced vision were also investigated with results indicating the ability of the multi-sensor cane to detect some changes from normal vision. Intuitively, the “both eyes closed” case was notably different. A lack of vision decreased the pitch velocity during swing and stance phases indicating that people walked slower when their vision was impaired. There was some evidence that users relied more on their cane when their vision was impaired but not occluded; however, no significant changes were observed. Stride length decreased significantly for the “both eyes closed” case indicating decreased walking confidence due to the loss of vision. Lastly, mean difference in IC event determined by MSMF with respect to GPD increased, indicating a change in walking pattern because of a change in vision. The ability to detect gait changes during impaired or occluded vision could be used to indicate unsafe walking conditions such as during the night without proper lighting. 

Finally, the results suggested that the multi-sensor cane is capable of identifying differences between cane lengths. Particularly, users tended to rely more on cane due to the improper cane lengths, and stride length increased and decreased in response to cane length with respect to the control case. The mean difference in IC, as determined by the MSMF and GPD approaches, increased for improper cane lengths, indicating a change in the walking pattern. Given these results, future work could seek to develop an automated cane length adjustment protocol that optimizes gait. 

Overall, the results presented here validate the ability of the multi-sensor cane to detect changes in gait due to the presence of simulated pain, walking terrains, perturbed vision, and improper setting of the cane. These results were validated using a shank-mounted IMU which provided results similar to those obtained by the cane. This is an important finding because, although the load through the cane is distributed through the upper body, it is still able to identify changes in gait caused due to the lower extremity. This strengthens the case for instrumented ADs as a deployable tool that could provide valuable information and feedback to their users, healthcare providers, and loved ones. 

## 5. Limitations and Future Work

Given the scope of this paper there are some limitations which future studies can address. The experiments in our current work were performed with young healthy participants. This decision was mainly motivated by the ease of recruiting university-going, young, healthy participants. The end goal of this research was to help individuals with disease or disabilities. Our ongoing subsequent work is seeking to extend current experiments with older adults and participants from clinical populations. Moreover, current experiments were focused to detect changes in gait due to the single condition confounding the measurement. Future studies are warranted to show the ability of multi-sensor canes to extract information when more than one confounding factors are present (i.e., walking downhill with pain in the heel, etc.). Lastly, it may be useful to characterize strides and evaluate the multi-sensor cane in terms of force by performing tests on balance/weight board. This knowledge may provide additional insights into capabilities of the multi-sensor cane. 

## 6. Conclusions

In this paper, a multi-sensor cane was used to detect changes from healthy walking patterns. Multi-sensor data were used to extract features that were compared between healthy walking conditions and a variety of perturbed walking conditions. The multi-sensor cane approach was found to be able to detect changes in gait that arose from changes in walking terrains such as walking uphill, downhill or up and down flights of stairs, differences in cane length, and impaired vision. That the low-cost, multi-sensor approach can detect changes in gait resulting from a variety of walking environments and conditions suggests that it could greatly assist in the preventative monitoring of gait. Future works should include monitoring the system’s ability to detect gradual changes over time in a longitudinal study.

## Figures and Tables

**Table 1:** Various walking conditions tested for each participant.

Condition	Detail
Control	Unperturbed cane-assisted 52 m walk on a flat surface. Participants moved the cane contralateral (opposite side) to the affected leg. This contralateral-side assistance was used for all conditions.
Dorsiflexion	Cane-assisted 52 m walk on flat surface. Participants were asked to dorsiflex to avoid putting any weight on the toes of their affected leg.
Plantarflexion	Cane-assisted 52 m walk on flat surface. Participant was asked to plantarflex to avoid putting any weight on the heel of their affected leg.
Upstairs	Unperturbed cane-assisted walk up 1 flight of stairs (20 steps).
Downstairs	Unperturbed cane-assisted walk down 1 flight of stairs (20 steps).
Uphill	Unperturbed cane-assisted 78 m walk on a paved sidewalk, uphill.
Downhill	Unperturbed cane-assisted 78 m walk on a paved sidewalk, downhill.
Fogged Glasses	Cane-assisted 52 m walk on a flat surface. Participant was asked to wear fogged glasses so that vision was impaired.
Both Eyes Closed	Cane-assisted 52 m walk on a flat surface while participant was blindfolded.
Long Cane	Cane-assisted 52 m walk on flat surface with cane adjusted to a length 2 inches longer than ideal.
Short Cane	Cane-assisted 52 m walk on flat surface with cane adjusted to a length 2 inches shorter than ideal.

**Table 2:** Various cane features tested for each condition.

Feature	Description
Max anteroposterior velocity (PV)—Swing Phase (°/s)	Maximum value of cane anteroposterior (AP) velocity measured in °/s during the swing phase.
Max PV—Stance Phase (°/s)	Maximum value of cane AP velocity measured in °/s during the stance phase.
Max PV Time Index with respect to TC (ms)	Time index of maximum value of cane AP velocity during the swing phase as measured with respect to the TC event time index in milliseconds.
Swing Stance Ratio	Ratio of swing phase to stance phase interval.
Max Strain (analog to digital converter (ADC) value)	Maximum value of strain (ADC value) applied to cane.
Max Strain Time Index with respect to IC (ms)	Time index of the maximum value of strain applied to the cane during the stance phase as measured with respect to the IC event time index in milliseconds.
Stride Length (m)	Calculated length of each stride measured in meters.
Difference in IC (ms)	Time difference in IC events from the cane data as measured by the multi-sensor matched filter (MSMF) and gyroscope peak detection (GPD) algorithms (i.e., IC_MSMF_ – IC_GPD_) measured in milliseconds.

**Table 3:** Features extracted from the cane sensors for simulated gait abnormalities (repeated measures ANOVA, F: F ratio, *p*: *p*-value); µ: mean value; σ: standard deviation; (results of the Bonferroni comparison are indicated by * control ≠ plant or dorsi and ** plant ≠ dorsi).

	ANOVA (F, *p*)	Control (µ ± σ)	Plant (µ ± σ)	Dorsi (µ ± σ)
Max PV—Swing Phase (°/s)	(5.2, <0.001)	(246.5 ± 55.5)	(228.3 ± 73.8) *	(204.9 ± 61.6) *, **
Max PV—Stance Phase (°/s)	(3.9, 0.03)	(156.4 ± 33.3)	(148.8 ± 28.5)	(124.6 ± 23.3) *, **
Max PV Time Index with respect to TC (ms)	(7.9, <0.001)	(258 ± 58)	(217.3 ± 52.4) *	(204.8 ± 35.9) *
Swing Stance Ratio	(0.8, 0.441)	(1 ± 0.2)	(0.9 ± 0.7)	(0.8 ± 0.2) *
Max Strain (ADC value)	(7.1, 0.002)	(110.6 ± 57.7)	(179.3 ± 93.3) *	(188 ± 83) *
Max Strain Time Index with respect to IC (ms)	(2, 0.141)	(361.9 ± 73.2)	(412.1 ± 99.9) *	(419 ± 80) *
Stride Length (m)	(1.3, 0.29)	(1.1 ± 0.4)	(0.9 ± 0.3) *	(0.8 ± 0.3) *, **
Difference in IC (ms)	(3.9, 0.024)	(−12.6 ± 6.6)	(−20.2 ± 11.6) *	(−25 ± 14.1) *

**Table 4:** Features extracted from the cane sensors for uphill and downhill walking (repeated measures ANOVA, F: F ratio, *p*: *p*-value); µ: mean value; σ: standard deviation; (results of the Bonferroni comparison are indicated by * control ≠ uphill or downhill and ** uphill ≠ downhill).

	ANOVA (F, *p*)	Control (µ ± σ)	Uphill (µ ± σ)	Downhill (µ ± σ)
Max PV—Swing Phase (°/s)	(7.1, 0.002)	(246.5 ± 55.5)	(270 ± 73) *	(268.1 ± 76.2) *
Max PV—Stance Phase (°/s)	(10, <0.001)	(156.4 ± 33.3)	(166.3 ± 27.5) *	(189.6 ± 33.5) *, **
Swing Stance Ratio	(0.1, 0.935)	(1 ± 0.2)	(1 ± 0.5)	(1 ± 0.3)
Max Strain (ADC value)	(1.8, 0.182)	(110.6 ± 57.7)	(159.7 ± 74.5) *	(135.7 ± 67.3) *, **
Stride Length (m)	(5.7, 0.005)	(1.1 ± 0.4)	(1.1 ± 0.4)	(1.2 ± 0.4) *,**
Difference in IC (ms)	(0.3, 0.722)	(−12.6 ± 6.6)	(−19.3 ± 12.8) *	(−12.5 ± 6.8) **

**Table 5:** Features extracted from the cane sensors for upstairs and downstairs walking (repeated measures ANOVA, F: F ratio, *p*: *p*-value); µ: mean value; σ: standard deviation; (results of the Bonferroni comparison are indicated by * control ≠ upstairs or downstairs and ** upstairs ≠ downstairs).

	ANOVA (F, *p*)	Control (µ ± σ)	Upstairs (µ ± σ)	Downstairs (µ ± σ)
Max PV—Swing Phase (°/s)	(158.4, <0.001)	(246.5 ± 55.5)	(68.3 ± 21.3) *	(79.6 ± 26.4) *, **
Max PV—Stance Phase (°/s)	(67.3, <0.001)	(156.4 ± 33.3)	(37.4 ± 8.8) *	(38.3 ± 14.2) *
Swing Stance Ratio	(5.7, 0.006)	(1 ± 0.2)	(0.7 ± 0.2) *	(0.7 ± 0.3) *
Max Strain (ADC value)	(3.2, 0.480)	(110.6 ± 57.7)	(201.7 ± 122.9) *	(179.8 ± 104.7) *
Stride Length (m)	(39.5, <0.001)	(1.1 ± 0.4)	(0.3 ± 0.2) *	(0.2 ± 0.1) *, **
Difference in IC (ms)	(17.3, <0.001)	(−12.6 ± 6.6)	(−78.7 ± 29.7) *	(−63.3 ± 27.4) *, **

**Table 6:** Features extracted from the cane sensors for impaired vision conditions (repeated measures ANOVA, F: F ratio, *p*: *p*-value); µ: mean value; σ: standard deviation; (results of the Bonferroni comparison are indicated by * control ≠ fogged glasses or both eyes closed and ** fogged glasses ≠ both eyes closed).

	ANOVA (F, *p*)	Control (µ ± σ)	Fogged Glasses (µ ± σ)	Both Eyes Closed (µ ± σ)
Max PV—Swing Phase (°/s)	(55.2, <0.001)	(246.5 ± 55.5)	(243.5 ± 63.6)	(194.8 ± 54.2) *, **
Max PV—Stance Phase (°/s)	(17, <0.001)	(156.4 ± 33.3)	(147.4 ± 24.9)	(104.1 ± 23.3) *, **
Swing Stance Ratio	(1.6, 0.202)	(1 ± 0.2)	(0.9 ± 0.2) *	(0.8 ± 0.3) *
Max Strain (ADC value)	(0.7, 0.524)	(110.6 ± 57.7)	(141.9 ± 80.9) *	(147.2 ± 76.3) *
Stride Length (m)	(9.3, <0.001)	(1.1 ± 0.4)	(1.1 ± 0.3)	(0.8 ± 0.3) *, **
Difference in IC (ms)	(3.1, 0.052)	(−12.6 ± 6.6)	(−19.3 ± 10.5) *	(−28.4 ± 15.9) *, **

**Table 7:** Features extracted from cane sensors for incorrect cane length conditions (repeated measures ANOVA, F: F ratio, *p*: *p*-value); µ: mean value; σ: standard deviation; (results of the Bonferroni comparison are indicated by * control ≠ long cane or short cane and ** long cane ≠ short cane).

	ANOVA (F, *p*)	Control (µ ± σ)	Long Cane (µ ± σ)	Short Cane (µ ± σ)
Max PV—Swing Phase (°/s)	(0.9, 0.392)	(246.5 ± 55.5)	(243.3 ± 61.7)	(246.9 ± 65.4)
Max PV—Stance Phase (°/s)	(1.3, 0.275)	(156.4 ± 33.3)	(146.9 ± 27.7)	(153.2 ± 28.3) **
Swing Stance Ratio	(1.2, 0.309)	(1 ± 0.2)	(0.9 ± 0.3) *	(1 ± 0.3)
Max Strain (ADC value)	(1, 0.363)	(110.6 ± 57.7)	(143.4 ± 77.7) *	(167 ± 85.1) *, **
Stride Length (m)	(18.8, <0.001)	(1.1 ± 0.4)	(1.3 ± 0.4) *	(0.8 ± 0.3) *, **
Difference in IC (ms)	(1.9, 0.165)	(−12.6 ± 6.6)	(−21.6 ± 10.2) *	(−23.3 ± 13) *

## References

[1] Florence C.S., Bergen G., Atherly A., Burns E., Stevens J., Drake C. (2018). Medical Costs of Fatal and Nonfatal Falls in Older Adults. J. Am. Geriatr. Soc..

[2] Wolff J.L., Starfield B., Anderson G. (2002). Prevalence, expenditures, and complications of multiple chronic conditions in the elderly. Arch. Int. Med..

[3] Houry D., Florence C., Baldwin G., Stevens J., McClure R. (2016). The CDC Injury Center’s Response to the Growing Public Health Problem of Falls Among Older Adults. Am. J. Lifestyle Med..

[4] Pavel M., Hayes T., Tsay I., Erdogmus D., Paul A., Larimer N., Jimison H., Nutt J. Continuous assessment of gait velocity in Parkinson’s disease from unobtrusive measurements. Proceedings of the 2007 3rd International IEEE/EMBS Conference on Neural Engineering.

[5] Yang G., Tan W., Jin H., Zhao T., Tu L. (2018). Review wearable sensing system for gait recognition. Clust. Comput..

[6] Hundza S., Hook W., Harris C. (2013). Accurate and reliable gait cycle detection in Parkinson ’s disease. IEEE Trans. Neural Syst. Rehabil. Eng..

[7] Yoneyama M., Kurihara Y., Watanabe K., Mitoma H. (2014). Accelerometry-based gait analysis and its application to parkinson’s disease assessment—Part 1: Detection of stride event. IEEE Trans. Neural Syst. Rehabil. Eng..

[8] Muro-de-la-Herran A., García-Zapirain B., Méndez-Zorrilla A. (2014). Gait analysis methods: An overview of wearable and non-wearable systems, highlighting clinical applications. Sensors.

[9] Akl A., Snoek J., Mihailidis A. (2017). Unobtrusive detection of mild cognitive impairment in older adults through home monitoring. IEEE J. Biomed. Health Inform..

[10] Wang K., Delbaere K., Brodie M., Lovell N.H., Kark L., Lord S.R., Redmond S.J. (2017). Differences between Gait on Stairs and Flat Surfaces in Relation to Fall Risk and Future Falls. IEEE J. Biomed. Health Inform..

[11] Rosenberg L., Kottorp A., Winblad B., Nygård L. (2009). Perceived difficulty in everyday technology use among older adults with or without cognitive deficits. Scand. J. Occup. Ther..

[12] Nygård L., Starkhammar S. (2007). The use of everyday technology by people with dementia living alone: Mapping out the difficulties. Aging Ment. Health.

[13] Demiris G., Rantz M.J., Aud M.A., Marek K.D., Tyrer H.W., Skubic M., Hussam A.A. (2004). Older adults attitudes towards and perceptions of ’smart home technologies: A pilot study. Inform. Health Soc. Care.

[14] Gill S., Hearn J., Powell G., Scheme E. Design of a multi-sensor IoT-enabled assistive device for discrete and deployable gait monitoring. Proceedings of the 2017 IEEE Healthcare Innovations and Point of Care Technologies (HI-POCT).

[15] Gill S., Nssk S., Seth N., Scheme E. Design of a smart iot-enabled walker for deployable activity and gait monitoring. Proceedings of the 2018 IEEE Life Sciences Conference (LSC).

[16] Wade J., Beccani M., Myszka A., Bekele E., Valdastri P., Flemming P., De Riesthal M., Withrow T., Sarkar N. Design and implem rumented cane for gait recognition. Proceedings of the2015 IEEE International conference on Robotics and Automation (ICRA).

[17] Dang D.C., Suh Y.S. (2018). Walking distance estimation using walking canes with inertial sensors. Sensors.

[18] Boyles R.W. (2015). Mechanical Design of an Instrumented Cane for Gait Prediction by Physical Therapists. Master’s Thesis.

[19] Culmer P.R., Brooks P.C., Strauss D.N., Ross D.H., Levesley M.C., Connor R.J.O., Bhakta B.B. (2014). An Instrumented Walking Aid to Assess and Retrain Gait. IEEE/ASME Trans. Mechatron..

[20] Gill S., Seth N., Scheme E. (2018). A multi-sensor matched filter approach to robust segmentation of assisted gait. Sensors.

[21] Ballesteros J., Urdiales C., Martinez A.B., Tirado M. (2017). Automatic assessment of a rollator-users condition during rehabilitation using the i-Walker platform. IEEE Trans. Neural Syst. Rehabil. Eng..

[22] Huang J., Di P., Wakita K., Fukuda T., Sekiyama K. Study of fall detection using intelligent cane based on sensor fusion. Proceedings of the 2008 International Symposium on Micro-NanoMechatronics and Human Science.

[23] Nahapetian A., Kaiser W., Au L., Sarrafzadeh M., Lan M., Vahdatpour A. SmartFall: An automatic fall detection system based on subsequence matching for the SmartCane. Proceedings of the Fourth International Conference on Body Area Networks.

[24] Sprint G., Cook D.J., Weeks D.L. Quantitative assessment of lower limb and cane movement with wearable inertial sensors. Proceedings of the 2016 IEEE-EMBS International Conference on Biomedical and Health Informatics (BHI).

[25] Kumar R., Roe M.C., Scremin O.U. (1995). Methods for estimating the proper length of a cane. Arch. Phys. Med. Rehabil..

[26] Mitschke C., Heß T., Milani L. (2017). Which Method Detects Foot Strike in Rearfoot and Forefoot Runners Accurately when Using an Inertial Measurement Unit?. Appl. Sci..

